# Finding the missing link

**DOI:** 10.7554/eLife.03128

**Published:** 2014-05-27

**Authors:** Thomas U Schwartz

**Affiliations:** 1 **Thomas U Schwartz** is at the Department of Biology, Massachusetts Institute of Technology, Cambridge, United Statestus@mit.edu

**Keywords:** membrane traffic, clathrin, TPLATE, TPC, muniscin, vesicle coat proteins, Dictyostelium, Human

## Abstract

The discovery of an ancient protein complex reveals the evolutionary relationships between the proteins that help to form vesicles.

**Related research article** Hirst J, Schlacht A, Norcott JP, Traynor D, Bloomfield G, Antrobus R, Kay RR, Dacks JB, Robinson MS. 2014. Characterization of TSET, an ancient and widespread membrane trafficking complex. *eLife* **3**:e02866. doi: 10.7554/eLife.02866

**Image** The full TSET complex contains six protein subunits.

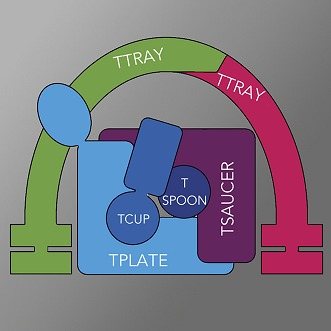

Eukaryotes, such as plants and animals, have an elaborate system of different compartments within their cells. These compartments, which are enclosed within membranes, include the nucleus; the endoplasmic reticulum, where many proteins are folded and modified; and the Golgi, where these proteins are sorted for delivery to other locations inside or outside the cell. Although these compartments provide specific environments that are best suited for particular tasks, they also create an enormous logistical problem: how can proteins be transported from one compartment to another?

Intense research over the past decades has revealed a number of distinct protein trafficking systems in eukaryotes, but we still do not fully understand how these different trafficking systems are evolutionarily related. Did they evolve separately, as their vastly different functions in the modern cell would suggest? Or did they diverge from a common ancestor, as certain similarities suggest? Now in *eLife*, Margaret Robinson of the University of Cambridge, Joel Dacks of the University of Alberta—together with co-workers in Cambridge, Alberta and the MRC Laboratory of Molecular Biology—reveal a previously undetected ancient relationship between the vesicle coat proteins that have a central role in different trafficking systems (Hirst et al., 2014).

Vesicles are the small, membrane-bound packages that traffic proteins between the different compartments in a eukaryotic cell. Three vesicle-trafficking systems have been widely studied, and are therefore the best understood: ‘clathrin-mediated endocytosis’ transports proteins from the cell's surface membrane to the inside of the cell; ‘COPII-mediated transport’ moves proteins from the endoplasmic reticulum to the Golgi; and ‘COPI-mediated retrotransport’ moves proteins from the Golgi back to the endoplasmic reticulum (D'Arcangelo et al., 2013; Faini et al., 2013; Kirchhausen et al., 2014; McMahon and Boucrot, 2011).

All three of these processes form vesicles by deforming a membrane into a curved pocket, but different vesicle coat proteins are used in the different systems. Adaptor protein complexes form an inner coat on the developing vesicle; they also directly interact with the membrane and help to select the cargo proteins that are packaged into the vesicle. An outer coat is then assembled on top of the adaptor protein layer, and forms a lattice-like framework that stabilises the vesicle.

Time after time, nature has been able to find a use for the new proteins that originate from random mutations of existing proteins: classic examples of this are the vast classes of enzymes that break down molecules of ATP and GTP in cells (Leipe et al., 2002; Iyer et al., 2004). Uncovering how all these enzymes were related to one another was aided greatly by the fact that they all contained certain sequences of amino acids. This strict conservation of key residues made it possible to detect other proteins that performed related jobs.

However, proteins that perform similar jobs do not always share a conserved sequence. In vesicle coating systems, for example, it is more important to conserve elements of 3D structure: this makes it much more difficult to detect related proteins.

One of the clearest signs of a common ancestor of specific coat proteins involved in the three vesicle-trafficking systems described above is that the five adaptor protein complexes involved in clathrin-mediated trafficking are structurally related to the adaptor protein component of the COPI coat (Hirst et al., 2013). These complexes all contain four protein subunits: two large subunits, a medium subunit, and a small subunit. Robinson, Dacks and co-workers—who include Jennifer Hirst and Alexander Schlacht as joint first authors—have now discovered a new adaptor protein complex that they call TSET (Hirst et al., 2014). This new complex has six subunits; and the sequences of these subunits are very different from those of the known adaptor proteins, which are already a remarkably sequence-divergent group of proteins.

In order to find TSET, Hirst, Schlacht et al. developed a powerful bioinformatics tool, called ‘reverse HHpred’. Typically, comparing the sequence of an unknown protein with alignments of sequences of proteins with known 3D structures can uncover proteins that have a similar shape (Söding, 2005). It turns out that doing the reverse, searching with a known 3D structure against appropriately curated datasets of the proteins of individual species, is an even more sensitive method (Kelley and Sternberg, 2009; Hirst et al., 2014). Searching datasets of the proteins from a range of different eukaryotes with the known structures of some adaptor proteins resulted in the detection of TSET components in most of groups of eukaryotes. However, a complete TSET (containing all six components) has been verified only in a plant (Gadeyne et al., 2014) and in a slime mould (Hirst et al., 2014). Most other organisms are predicted to have only a subset of these six proteins. As such, it is likely that the last common ancestor of all eukaryotes contained the complete TSET complex, and that individual components have been lost independently in different organisms over the course of approximately two billion years of evolution.

The new data support the idea that the various vesicle-coating complexes within eukaryotic cells are distantly related. That said, the differences between the systems are remarkable. For example, outer coat proteins found in COPI and clathrin are only very superficially related and seem to assemble in entirely different ways (Faini et al., 2013). Therefore, the evolution of the modern vesicle coating systems appears to have involved adapting some building blocks derived from a common ancestor, and adding new proteins in each of the different systems. The ‘reverse HHpred’ method will now help researchers to find more of the distant relatives of highly divergent proteins and improve our understanding of the evolutionary relationships between different proteins in general.
